# Chronotype and response to training during the polar night: a pilot study

**DOI:** 10.1080/22423982.2017.1320919

**Published:** 2017-05-19

**Authors:** Jacopo Antonino Vitale, Eva Bjoerkesett, Andrea Campana, Giacomo Panizza, Andi Weydahl

**Affiliations:** ^a^ Laboratory of Structures Mechanics, IRCCS Istituto Ortopedico Galeazzi, Milan, Italy; ^b^ School of Sport Sciences, UiT The Arctic University of Norway, Alta, Norway

**Keywords:** Circadian typology, darkness, eveningness, health, latitude, MEQ, morningness, sport, polar circle

## Abstract

**Background**: An individual’s chronotype influences his or her physiological rhythms. Some studies have looked at the effect of time of day on the responses to exercise, but studies on the effect of long-term training are lacking.

**Objective**:  To report the effects of an 8-week training period during the polar night in non-athletes of different chronotypes living at 70°N.

**Design**:  In all, 10 morning (M), 10 neither (N) and 10 evening (E) types were recruited, and their aerobic capacity (VO_2max_), strength, flexibility and balance before and after the training period were tested.

**Results:** 3 E-types, 5 N-types and 6 M-types completed the protocol. An increase in VO_2max_ and strength was observed for the whole group. The best negative correlation (r=–0.5287) was found between the Morningness–Eveningness Questionnaire (MEQ) score and the increase in VO2max, and the best positive correlation (r=0.4395) was found between MEQ and the increase in strength. Changes in balance and flexibility did not show any clear trends.

**Conclusion:** In an environment with no outdoor daylight, it seems that the response to 8 weeks of aerobic training is larger in the E- than in the M-types, although the M-types showed a larger improvement in strength.

## Introduction

Modern society is based on a 24-hour day, with the social rhythm divided between work and leisure. In addition to this social rhythm, everyone has his or her own biological or circadian rhythm, determined by various hormones. A person’s circadian rhythm (an approximately 24-hour rhythm) is rarely consistent with the social 24-hour rhythm. It can be a bit less or a bit more than 24 hour (e.g. 23 or 25 hours) and is based on when the person is most alert and active. Such rhythms in different people are known as different chronotypes [1]. There are morning people (M-types) who are most active and alert in the morning and evening people (E-types) who are most active and alert in the evening [2]. A third group is the neither type (N-type) because they are neither E- nor M-types. This circadian typology is involved not only in habits and lifestyles, emerging in particular during adolescence and remaining present throughout adult life, but also in the expression of physiological rhythms [3]. Circadian differences in physiological parameters could lead to different physical performance throughout the day, as several studies have shown [4–6].

The northern part of Norway is above the Arctic Circle (about 66°33′N). This region is special because there is a period of the year when the sun does not rise above the horizon (polar night) and a similar period when the sun does not descend below the horizon (midnight sun) [7]. No other area in the world has a population living in such an environment with either no light or constant outdoor light. These large fluctuations in amount of light, and the time at which the organs of the body are influenced by light, have been shown to have a direct effect on an organism’s functions [8] and responses to stimulation [9].

Physical fitness is an important element of people’s health, and the promotion of greater levels of physical fitness in the population is an important goal in public health [10]. To maintain or achieve good physical fitness, it is necessary to be physically active and to carry out physical training or exercise. To achieve and improve physical fitness, and therefore to have a training effect, it is necessary to stress the body with an increased load that lasts for a while. The size of the training effect depends on the size of the load, the duration of the stress, the repetition and the person’s physical fitness at the start of the exercise [11]. Referring to all these aspects of an individual’s physical fitness, it is crucial to emphasise that the chronotype could significantly influence both the psycho-physiological responses to a physical task and the person’s predisposition or attitude towards physical activity and an active lifestyle. It has been shown that E-types are more likely to smoke tobacco, to spend more hours watching television and, most of all, to have generally sedentary behaviour [12,13]. Against this, a healthier lifestyle has been observed in M-types, which could be partially explained by the chronotype differences in personality factors: morningness is associated with conscientiousness, including lower levels of procrastination [14], and with a higher volume of habitual physical activity than E-types [15]. With regards to the chronotype effect on physical performances, in earlier projects, it was shown that different chronotypes reacted differently to the same exercise: 46 individuals (27 N-, 10 E- and 9 M-types) performed a physical task consisting of self-paced walking up and down a hill three times, in both the morning (08:30) and the afternoon (16:30). It was observed that the rate of self-perceived exertion (RPE) was significantly higher in E-types (14.33 [SD=2.45]) compared with M-types (12.00 [1.66]) after the morning session (p=0.01, Cohen’s d=1.10) [16,17]. Other recent studies have examined the chronotype effect on physical performance: Rae et al. [18] compared a 200-metre swimming time trial performed at 06:30 and 18:30 in 26 trained swimmers classified as 15 M-types and 11 N-types. The authors reported that M-types swam faster in the morning session and N-types swam faster at 18:30 (p=0.036). Swimmers with higher Morningness–Eveningness Questionnaire (MEQ) scores, who therefore had a strong predisposition towards morningness, tended to swim faster at the morning session (p=0.025). In addition, Facer-Childs and Brandstaetter [19] observed ﬁeld hockey players (5 M-, 10 N- and 5 E-types) who performed the bleep test (also known as the multi-stage ﬁtness test) at six different times of the day (07:00, 10:00, 13:00, 16:00, 19:00 and 22:00) and found that the highest performance occurred at 12.19±1.43 hours for M-types, 15.81±0.51 hours for N-types (p<0.05) and 19.66±0.67 hours for E-types.

It has also been highlighted the fact that, by omission of the chronotype effect, the response to the same exercise load differed if the activity was carried out in the morning rather than the evening: female participants reached a higher peak heart rate (HR) when they trained in the evening versus the morning (p=0.002, Cohen’s d=3.04) [20,21]. Physical performance and response to training stimuli have been shown to change with time of day [21]. As the individual’s chronotype influences the expression of physiological rhythms, it could influence the response to training stimuli and affect athletic performance at various times of day [22]. However, no previous studies have looked at responses to longer training periods with major differences in outdoor lighting conditions, or at the training of different chronotypes.

In all rehabilitation and physical training, it is important to find the right training load for each athlete or patient [23]. Too high a load could cause injury, whereas too low a load will not produce the desired development. Without the correct training load, it takes longer for a positive effect, if any, to be seen, which could lead to a lack of motivation [24] and participants stopping their training. Time and money will have been spent to no positive effect, the health impact of increased efforts will have failed to materialise and the resources used could have been better spent.

The aims of the present study were: (1) to look at the changes in physical fitness, expressed as maximal oxygen consumption (VO_2max_), flexibility, strength and balance, in untrained adults with different circadian preferences (chronotypes) who live at 70°N when they train during the polar night (in this study, this was from 26 November to 17 January); and (2) to evaluate the adherence to the experimental protocol in M-, N- and E-types.

Based on the scarce literature in this field, the following hypotheses are proposed:

1. The chronotype will have an effect on the improvements in VO_2max_, flexibility, strength and balance after 8 weeks of training during the polar night. Specifically, E-types will show larger and better increases for all variables in the post-test condition compared with M-types because all group training was carried out after 19:00, a time of day that should favour the E-types. In addition, the environment without constant outdoor light will have a negative effect on the results for M-types.
2. The chronotype will have an effect on adherence to the experimental protocol; specifically, M-types will show greater participation in the training protocol compared with E-types.

## Methods

An interventional, pre-testing–training–post-testing protocol was used in the present study.

### Participants

A newspaper and internet invitation to determine individuals’ chronotypes using Horne and Östberg’s [25] MEQ resulted in 262 individuals being interested in participating in the project. From their results on the MEQ, the participants were categorised as 114 E-types (scores between 16 and 41), 102 N-types (scores between 42 and 58) and 46 M-types (scores between 59 and 86), according to the MEQ rating scale [25].

The participants had to be healthy and not actively competing athletes, and they had to stay in the area with the polar night for the whole of the intervention, so some of the interested participants had to be excluded. The exclusion criteria were: having a nocturnal part-time job; age <18 years or >50 years; having any medical conditions that contraindicated physical exercise, as established by a sports medicine specialist; body mass index (BMI) >40; having participated and trained in competitive sports for up to 4 years before the project; and planning to leave the region during the polar night period.

For practical reasons, such as size of training group and time needed for testing, we could include only a total of 30 participants in the intervention.

**Figure 1.**
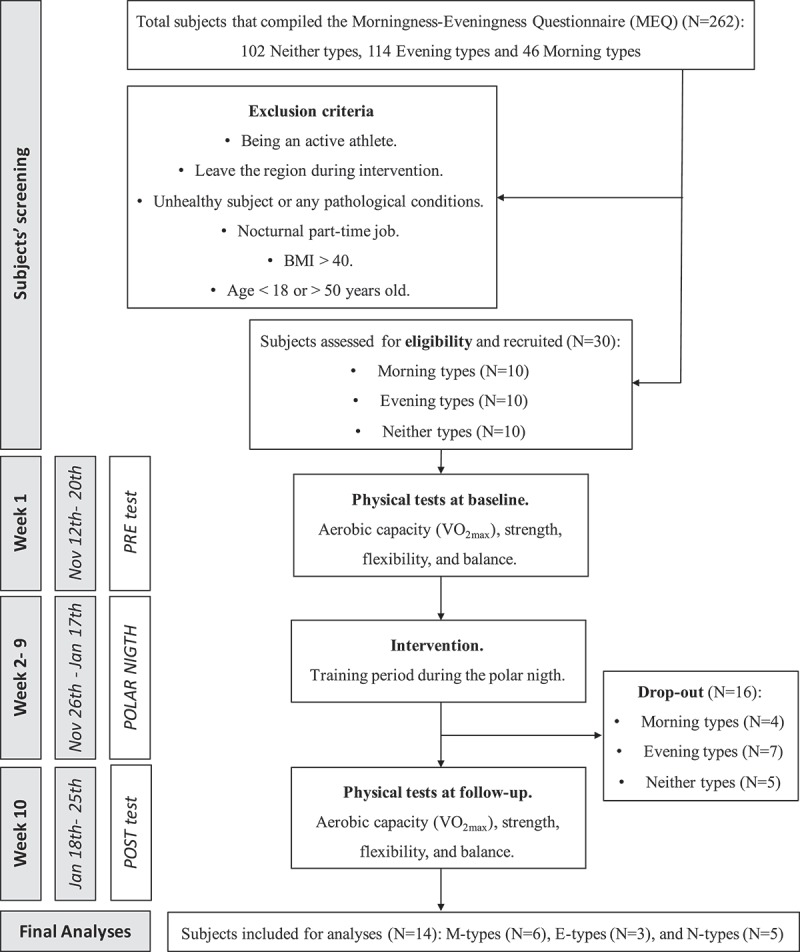
Flowchart for the study design and the subject’s screening/selection according to the inclusion and exclusion criteria.

After exclusion of those who were not eligible to participate, the 10 individuals with the lowest MEQ score of <41 (E-types), the 10 with the highest MEQ score of >59 (M-types) and the 10 with an MEQ score closest to 50 (N-types) were chosen to participate. We ended up with: 10 E-types: MEQ 22–30 (five females and five males); 10 N-types: MEQ 46–52 (seven females and three males); and 10 M-types: MEQ 63–77 (seven females and three males). Figure 1 illustrates the study flowchart.

The participants received an explanation of the project’s aims, methods and possible risks. They all signed a written informed consent form in order to participate in the study, which was approved by the Ethical Committee of the Norwegian Social Science Data Service (project no. 40015), which complies with current international laws and regulations governing the use of human participants (Declaration of Helsinki II).

### Measures and procedures

The MEQ is composed of 19 questions with four or five multiple-choice answers, each answer having a different score (between a minimum of 0 and a maximum of 6). The reliability of the internal consistency and the test–retest, as well as the external validity of the MEQ, have been reported in previous studies [26,27]. The total range for the MEQ score is 16–86, and all of the questions refer to the participant’s personal sleep habits. One representative question is given by item 4: “How easy do you find it to get up in the morning (when you are not awakened unexpectedly)?” This question has the four answers: (1) very difficult; (2) somewhat difficult; (3) fairly easy; and (4) very easy.

On the last week before the start of the polar night (12 November–20 November) the participant’s VO_2max_ values were tested using direct measurement with an incremental protocol on a Woodway Desmo HP treadmill, walking or running at speed, with a gradient set according to the individual’s familiarity with working on a treadmill and assumed fitness. A mixing chamber system from Oxycon Pro (Erich Jaeger GmbH, Hoechberg, Germany) was used for gas analyses. As a warm-up, the participants started for 10 minutes at 8 km/hour on a 0–5% incline; thereafter, workloads of 5 minutes with lactic acid sampling were added, until the individual reached a lactic acid level of 4 mmol/ml. The participant had a short rest period and started the incremental test for VO_2max_, running at 8 km/hour on a 5% incline for 4 minutes, then increasing the speed by 1 km/hour every 30 seconds until the respiratory exchange ratio exceeded 1.1; then, the speed was maintained until exhaustion or a levelling off or decrease of the VO_2_.

Those who were not familiar with running on the treadmill performed a “Balke test”: walking for 4 minutes at 4.8 km/hour on a 4% incline, with the incline increasing thereafter by 2% every minute up to 20%, and after that the speed increased by 0.5 km/minute until exhaustion. After the VO_2max_ test, we tested static back extension, one-leg standing, handgrip using a dynamometer (from Chattanooga Medical Supply, Inc., Chattanooga, TN, USA), modified push-ups, sit and reach and back scratch. The protocols used were the same as those used in the Norwegian Fitness Survey [28] and were recommended by the international expert group Assessing Levels of Physical Activity and Fitness (ALPHA) [29]:

- One-leg standing: blindfolded if longer than 60 seconds.
- Sit and reach: hamstring flexibility (sitting with straight knees and soles on a plate, measuring how far the middle fingers reach on a centimetre scale starting on 23 cm for the soles).
- Back scratch: shoulder flexibility (distance between fingertips when trying to get right and left hands to meet on the back, one hand coming from above and the other from below the shoulder; to avoid negative values because of the fact that many individuals did not have fingertips overlapping, we chose to add 25 to the measured centimetre value).
- Static back strength: how long the person can keep the back horizontal while lying on a bench with no support for the hips and a helper sitting on the thighs.
- Upper body strength: number of modified push-ups in 40 seconds, starting lying on the floor with hands on the hips, moving hands to shoulders and pressing up with straight back/hips to straight arms, moving one hand to touch the other hand and returning down to lying position with hands on hips.
- Grip strength: best results of three efforts at pressing the hydraulic dynamometer.

The week after the polar night ended (18–25 January), the participants were retested (post-test) using the same tests as for the pre-test.

During the polar night period, the participants had to do home-based exercise every day, such as running or walking outside for at least 10 minutes, and strength exercises (lying down on the floor – raising up 10 times, 10 push-outs in a corner, a sit-up programme, 10 hand–knee stands, stretching the opposite arm and leg, 10 long steps forward, both legs first), flexibility (using a short towel when drying the back) and balance (standing on one leg while brushing teeth). Every day, they ticked off on their training programme every exercise that they had performed that day. In addition, they were invited to two 1-hour group training sessions every week, one indoors and one outdoors. The inside programme (Tuesday at 20:00) consisted of a warm-up and a circular programme of four rounds with 40 seconds of activity at eight stations:

1. Balancing back and forth on a bench that was 20 cm high, 10 cm wide and 250 cm long, doing moves to challenge balance;
2. Brisk walking with high-knee lifts;
3. Sitting with bent knees and moving a 3-kg ball from side to side;
4. Standing back to back with a partner, one with a 3-kg ball, holding it with straight arms in front, lifting the ball and handing it over the head to the partner, who lowers the ball and lifts it up to return it to the person;
5. Standing at a rib-wall, with the front to the wall, using the arms just to hold the position and the ankles to lift and lower the body;
6. Plank;
7. Running on a high-jump mat;
8. Push-ups.

The outdoor programme (Thursday at 19:00) was used for aerobic training and consisted of, for example, 2 km of fast walking and going up and down a steep hill three times for 3 minutes, as well as short-interval training and pyramidal intervals.

The HR was recorded using a PolarTeam2 device (Polar Electro Oy, Kempele, Finland). During the supervised exercise, the participants also used watches that showed their HR during training. The participants had the option to use HR monitoring belts during the home-based exercise. Those who used these HR belts handed in the recorder every week. The recorded HRs were printed out and given to the participants. In this way, we planned to control the participants’ work intensity in order to inform and educate them about their work intensity during training.

For the home-based exercise, they had a programme to follow. After performing each exercise, they had to tick it off on their daily log and make notes about any extra exercise.

### Treatment of data

We calculated the changes in the test results from pre-test to post-test as the percentage change from the pre-test ([{post-test score – pre-test score}/pre-test score] × 100) for each variable tested. As strength and flexibility were tested with more than one exercise, we created two indices in order to express strength and flexibility: the weighted mean for our participants was calculated using averages from a large sample from north Norway [28], separated by gender. We calculated the reciprocal of the mean and multiplied this by 100 in order to find an index for the different exercises.

Scatter plots with trend lines were created in order to obtain an overview of possible connections between MEQ scores and background variables and training and fitness variables, as well as to check for obvious outliers. Participants who did not take part in group training or the post-test evaluation were not included in the data analyses.

### Analyses

Mean values for background variables, as well as fitness variables, were calculated for both pre- and post-tests.

The distributions of VO_2max_, strength and flexibility indices and balance in the pre-test and post-test conditions for the whole sample were tested for normality using the Shapiro–Wilk test. All of the parameters were normally distributed with the exception of balance in all conditions and the strength indices in pre-test conditions.

Simple scatter plots between MEQ scores and each of the fitness variables, as well as their percentage changes from pre-test scores, were made, including trend lines with formulae and r^2^. Pearson’s correlations were also run between MEQ scores and the percentage changes from the pre-tests of each of the fitness variables. We used Microsoft Excel 2013 for all data treatment.

## Results

Of the 30 participants at the start of the study, 16 did not participate in the post-test evaluation. From the scatter plot of the VO_2max_ data versus MEQ score and the scatter plot of the sit-and-reach data versus MEQ score, we found some data that we considered to be outliers: one M-type participant because of the large increase in VO_2max_ (58%) and one E-type with a large increase in sit-and-reach data (133%). These data were not included in the calculation of mean values (VO_2max_ ID: 12; sit-and-reach ID: 2). In total, three E-types, five N-types and six M-types were included in the study (n=14: six men, mean age=33 [SD=9.2] years; eight women, mean age=39 [7.5] years). Tables 1 and 2 present the raw anthropometric data and all variables measured for each participant before and after the training period.

**Figure 2.**
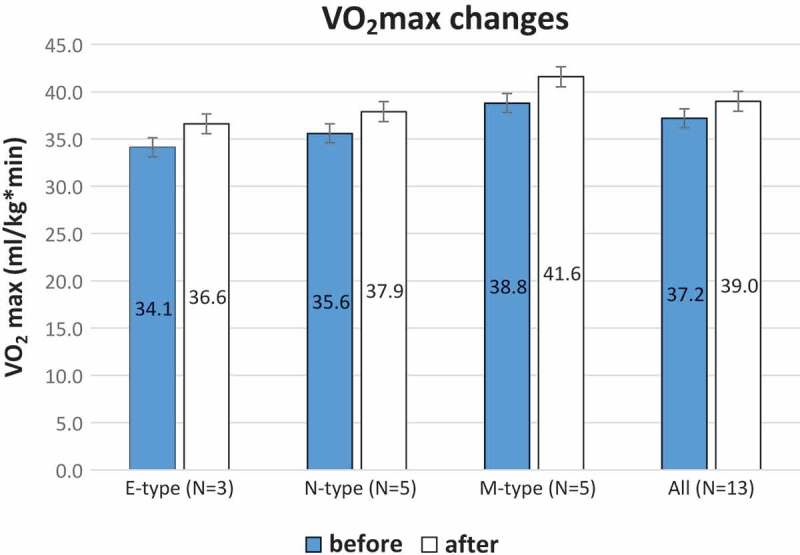
Mean values with standard errors of VO_2max_ in ml/kg per minute before (blue histograms) and after (white histograms) the period of training for E-types (n=3), N-types (n=5) and M-types (n=5), and all participants with valid VO_2max_ results (n=13).

**Figure 3.**
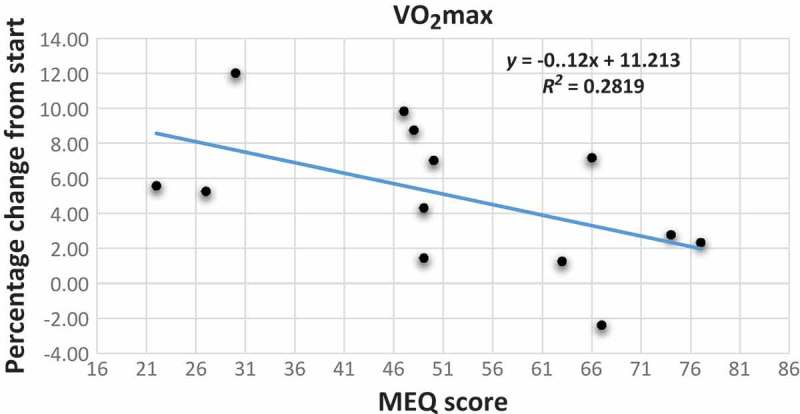
Scatter plot with trend line showing the relationship between the percentage change from start of VO_2max_ as the dependent variable and the MEQ scores of the participants. MEQ score scale: E-type 16–41; N-type 42–58; M-type 59–86.

Figure 2 is a graphic illustration of the increase in VO_2max_ for the three groups of chronotype and for the whole sample. From the scatter plot with the trend line between MEQ scores and the improvement in VO_2max_ values, as reported in Figure 3, we observed that participants with higher MEQ scores, showing a strong predisposition towards morningness, tend to have a smaller increase in VO_2max_ than those with lower MEQ scores (*y*=–0.12*x*+11.213; r^2^=0.279).

**Figure 4.**
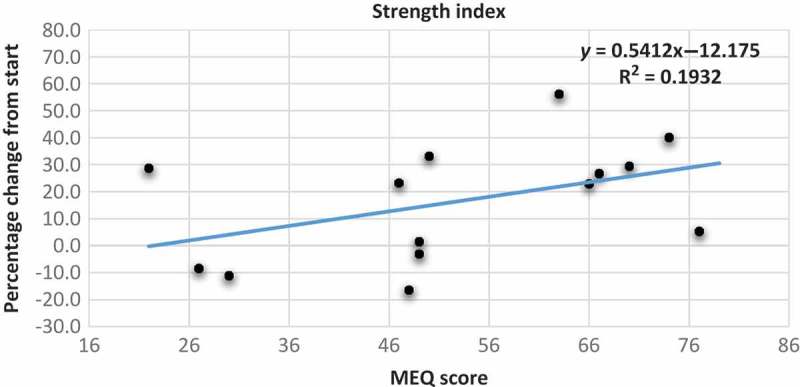
Scatter plot with trend line showing the relationship between the percentage change from start of strength index as the dependent variable and the MEQ scores of the participants. MEQ score scale: E-type 16–41; N-type 42–58; M-type 59–86.

**Figure 5.**
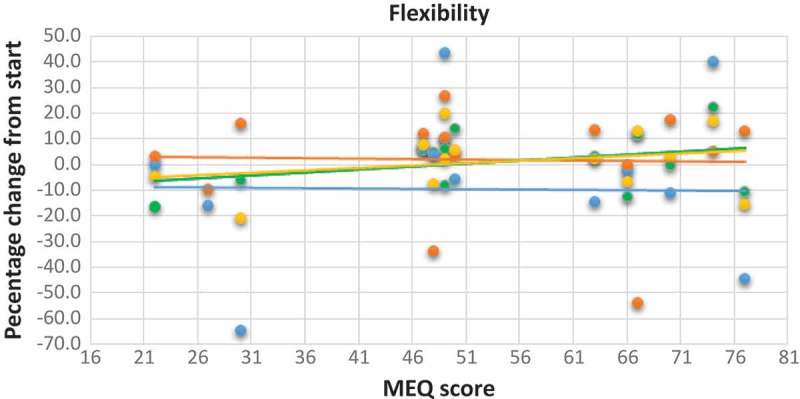
Scatter plot with trend line showing the relationship between the percentage change from start of the different flexibility tests as the dependent variable and the MEQ scores of the participants. MEQ score scale: E-type 16–41; N-type 42–58; M-type 59–86. Blue dot and line: percentage change for left side; orange dot and line: percentage change for right side; green dot and line: percentage change in sit and reach test; yellow dot and line: percentage change in flexibility index.

**Figure 6.**
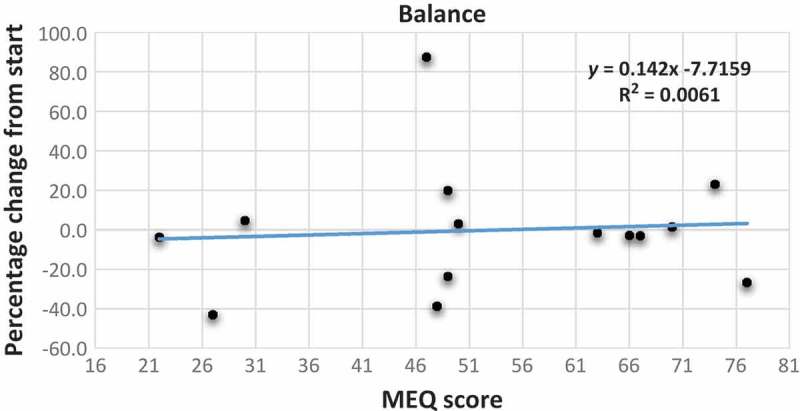
Scatter plot with trend line showing the relationship between the percentage change from start of balance as the dependent variable and the MEQ scores of the participants. MEQ score scale: E-type 16–41; N-type 42–58; M-type 59–86.

Figure 4 shows the scatter plot with the trend line between MEQ scores and change in strength indices. We observed that participants with higher MEQ scores tend to have a larger increase in their strength index than those with lower MEQ scores (*y*=0.5412*x*–12.175; r^2^=0.1932). In addition, from the scatter plots with the trend line between MEQ scores and change in flexibility scores and balance scores (Figures 5 and 6), there was no connection with the MEQ score. This was confirmed with Pearson’s correlations (Table 3).

**Table 1.** Raw data for all the single participants (ID no.) before (pre) and after (post) the period of training.

					BMI		VO_2max_		Strength index		Flexibility index		Balance (seconds)	
	ID no.	MEQ score	Age	Sex	*Pre*	*Post*	*Pre*	*Post*	*Pre*	*Post*	*Pre*	*Post*	*Pre*	*Post*
E-types	1	22	30	Female	28.1	28.6	31.5	33.26	179	231	275	262	80	77
	2	27	24	Male	22.1	22.6	41.8	44	294	269	202*	212*	100	57
	8	30	49	Female	29.8	28.3	29.1	32.6	184	164	364	289	63	66
N-types	4	47	42	Female	23	23.6	32.7	35	270	333	323	348	64	120
	5	48	26	Male	23	23.1	47.7	52.4	425	355	243	225	106	65
	6	49	46	Female	33.8	32.3	28.5	31	218	212	196	235	68	52
	7	49	25	Male	35.5	35.5	34.3	34.8	257	261	354	363	65	78
	3	50	45	Female	23.7	23.4	34.8	36.3	256	342	248	261	65	67
M-types	9	63	31	Female	23.3	22.4	39.5	40	223	349	283	290	67	66
	10	66	32	Female	30.3	30.3	39	41.8	220	271	237	221	70	68
	11	67	43	Male	30.6	30.7	33.5	32.7	260	329	73	83	66	64
	12	70	36	Male	33.8	31.9	29.6*	47*	294	381	207	213	65	66
	13	74	44	Male	31.3	31.6	39.6	40.7	273	383	148	174	65	80
	14	77	38	Female	19.3	19.4	51.6	52.8	424	447	296	251	120	88

MEQ score scale: E-type 16–41; N-type 42–58; M-type 59–86. *V*O_2max_ is in ml/kg per minute. Strength and flexibility indices were used to consider together three different exercises. *Not included in the calculations.

**Table 2.** Raw data for all the single participants (ID no.) for different flexibility tests before (pre) and after (post) the period of training.

				Left above (cm)		Right above (cm)		Sit and reach (cm)		Flexibility index	
	ID no.	Age (years)	Sex	*Pre*	*Post*	*Pre*	*Post*	*Pre*	*Post*	*Pre*	*Post*
E-types	1	30	Female	25	25	30	31	25	21	275	262
	2	24	Male	25	21	31	28	6*	14*	202*	212*
	8	49	Female	38	13.5	25	29	41	38.5	364	289
N-types	4	42	Female	29	31	28,5	32	35	37	323	348
	5	26	Male	26	27	24	16	21	22	243	225
	6	46	Female	15	21.5	15	19	25	26.5	196	235
	7	25	Male	30	33	33,5	37	38	35	354	363
	3	45	Female	18	17	33,5	35	21	24	248	261
M-types	9	31	Female	21	18	29	33	31	32	283	290
	10	32	Female	18.5	18	20	20	28.5	25	237	221
	11	43	Male	–5	–1	6.5	3	17	19	73	83
	12	36	Male	9	8	17	20	31	31	207	213
	13	44	Male	5	7	19	20	18	22	148	174
	14	38	Female	31.5	17.5	26.5	30	28	25	196	251

MEQ score scale: E-type 16–41; N-type 42–58; M-type 59–86. Left above: shoulder flexibility left arm above; right above: shoulder flexibility right arm above; sit and reach: hamstrings flexibility; flexibility index: calculated score based on the tested values; see “Methods” section.

*Not included in the calculations.

**Table 3.** Results from Pearson’s correlation analysis between the MEQ score (MEQ score scale: E-type 16–41; N-type 42–58; M-type 59–86) and changes in fitness variables after 10 weeks of training during polar night period at 70°N, expressed as percentage of change from pre-test score.

Variables associated with MEQ score	Pearson’s correlation index
Percentage change in VO_2max_	–0.5287
Percentage change in strength index	0.4395
Percentage change in flexibility index	0.2667
Percentage change in flexibility (right arm above)	–0.0348
Percentage change in flexibility (left arm above)	–0.0127
Percentage change in flexibility (sit and reach)	0.3372
Percentage change in balance	0.0784

**Figure 7.**
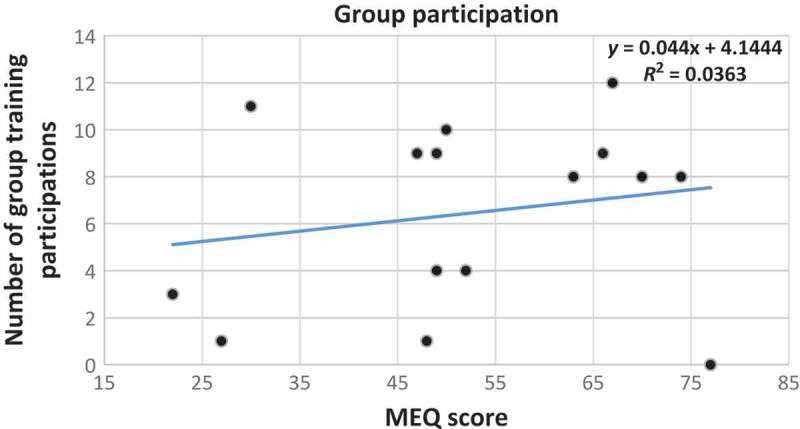
Scatter plot with trend line showing the relationship between the number of participants attending group training as the dependent variable and the MEQ scores of all of the participants.

With regards to the scatter plot, with the trend line between MEQ scores and the number of attended group training sessions, as reported in Figure 7, we did not detect a significant correlation. We just noted that those with higher MEQ scores tend to participate slightly more in group training than those with lower MEQ scores (*y*=0.044*x*+4.1444; r^2^=0.036), even if the relationship between these two variables was not strong.

## Discussion

The hypotheses stated at the start of the study were only partially confirmed. With regards to adherence to the training programme during the polar night, even though we started with a reasonable number of participants – 10 for each chronotype – there were few who reached the end of the programme. In particular, the pilot study ended up with very low numbers for E-types and N-types (three and five, respectively), although only four M-types quit the training programme (six of ten reached the end). This was an expected result. It has been demonstrated that M-types are more conscientious and stable compared with E-types [1], and conscientiousness, which is considered to be the strongest personality predictor of diurnal preference [30], has been positively associated with habitual physical activity [15]. Furthermore, it has been demonstrated that eveningness is a risk factor for a number of unhealthy habits [31], such as physical inactivity [32], and E-types also show greater daytime sleepiness [33]. These personality and behaviour characteristics, in relation to chronotype, may have led to a lower number of drop-outs among morning participants. In the present study, it seems that the higher the MEQ score (the more extreme M-type), the more participation there was in training, but a larger group of participants is needed in order to understand this is more detail. Lastly, as mentioned previously, the M-types are the group with the fewest drop-outs.

With reference to the changes in physical fitness, the participants, as a group, significantly increased their VO_2max_ values and strength indices. An outlier among the M-types, who showed an incredible increase in his VO_2max_ (58%, with a 1.9 decrease in BMI), was found and excluded from the analysis. There were some interesting points with regards to different chronotype groups: first, M-types did not increase their VO_2max_ unlike E-types; morning participants tended to have a smaller increase in VO_2max_ compared with E-types (r=–0.5287). Second, M-types had a larger increase in the strength index than those with lower MEQ scores, which refers to the participants with a predisposition towards eveningness (*r*=0.4395). On the other hand, the changes in balance and flexibility did not vary in relation to chronotype.

These findings are probably influenced by the small sample size and our initial hypothesis that E-types – those with lower MEQ scores – would have shown larger increases in all the examined variables compared with M-types, being only partially confirmed in the post-test condition. M-types are called M-types because they are more active in the morning [1,5], so as all group training sessions were carried out after 19:00 (a time that could favour E-types), they should have been more tired and less motivated to perform physical activity late in the day. It was shown by Rossi et al. [17] that E-types reported a higher RPE than M-types when exercising in the morning. This could mean that E-types do not train in the morning with the intensity necessary for a training effect because of a strong feeling of exertion. As our training sessions were carried out at 19:30, we wonder whether the training time could lead to a higher RPE among the M-types with low training intensity, one that is not sufficiently high to achieve the expected training effect [11]. If participants felt more tired at lower intensities, the HR might not reach values that produced increased endurance. The perception of exertion was not considered in the present study. In further studies, it will be necessary to include the RPE and compare it with the HR during the training sessions.

Finally, we hypothesised that the environment with no constant outdoor light would have a negative effect on the results for M-types and give an advantage to E-types because they are used to being more active at times of the day in which there is less outdoor light. Nevertheless, it is essential to highlight that there is no literature in this area and, for this reason, we are convinced that further studies are needed in order to postulate a new accurate hypothesis on the influence of long-term training during the polar night.

A good point of the study is that the PolarTeam2 device was used for all participants, which is a very accurate way of viewing their efforts and monitoring how much time they spent in each intensity zone. This point is also interesting in order to understand which type of chronotype was more motivated to do physical exercise at this particular time of the year. Unfortunately, we could not perform analyses of the HRs because of a lack of data. In future studies, it would be necessary to provide a better explanation to participants about the importance of this part of the study.

In further research, it would also be an idea to use a control group with the same chronotype characteristics, but without the period of training in the months between the tests. This would strengthen the assumption that the changes in the experimental group arose from the difference in response to training between different chronotypes during the polar night. In conclusion, it will be interesting to see whether and how training in the morning rather than the evening, and during daylight, might have an influence on the training effect in different chronotypes.

### Limitations of the study

The experimental protocol of this study had a number of limitations. First, the small numbers of participants for each chronotype category, in particular the lack of extreme M- and E-types, suggest that it is necessary to add further studies examining a larger group of participants. We think that this pilot study should be used to determine the correct sample size for a future study. Another limitation is the fairly large age range of the participants (24–49 years), and also the sample being made up of untrained males and females. It would be advisable, for future studies, to select a much more homogeneous sample for age, gender and background information in order to avoid possible bias in the results, such as by recruiting only trained male college students.

It would also be useful to select more specific physical tests, with the aim of detecting much larger and more accurate differences among chronotypes in the post-test condition (i.e. a short anaerobic physical test). In addition, the training protocol during the polar night should be standardised for the function of the selected physical test and given to all participants at the same time more reliably. Furthermore, compliance with the training sessions should be evaluated when the data are analysed.

As the clearest result in the literature can be observed for RPE and fatigue among chronotypes in relation to physical activity, it would also be good to detect the RPE during the whole period of training in darkness, observing possible differences in M-, E- and N-types.

In further studies, strict protocols using the right chronobiological and methodological approaches should be strongly considered, including controlled diet and environmental conditions, which are factors that could significantly influence sports performances and, consequently, the effect of chronotype.

## Conclusion

In this small pilot study with untrained adults living at 70°N, it was found that E-types tended to have a larger increase in VO_2max_ compared with M-type participants after training for 8 weeks during the polar night. M-types, however, had a larger increase in the strength index than E-types. No influence of chronotype was observed on flexibility and balance.

## References

[1] Adan A, Archer SN, Hidalgo MP, et al Circadian typology: a comprehensive review. Chronobiol Int. 2012;29:1153–11. 23004349 10.3109/07420528.2012.719971

[2] Vitale JA, Roveda E, Montaruli A, et al Chronotype influences activity circadian rhythm and sleep: differences in sleep quality between weekdays and weekend. Chronobiol Int. 2015;32:405–415. 25469597 10.3109/07420528.2014.986273

[3] Baehr EK, Revelle W, Eastman CI. Individual differences in the phase and amplitude of the human circadian temperature rhythm: with an emphasis on morningness–eveningness. J Sleep Res. 2000;9:117–127. 10849238 10.1046/j.1365-2869.2000.00196.x

[4] Drust B, Waterhouse J, Atkinson G, et al Circadian rhythms in sports performance – an update. Chronobiol Int. 2005;22:21–44. 15865319 10.1081/cbi-200041039

[5] Roveda E, Vitale JA, Montaruli A, et al Predicting the actigraphy-based acrophase using the Morningness-Eveningness Questionnaire (MEQ) in college students of North Italy. Chronobiol Int. 2017:1–12. [Epub ahead of print]. 10.1080/07420528.2016.1276928 28276851

[6] Vitale JA, Bonato M, Galasso L, et al Sleep quality and high intensity interval training at two different times of day: a crossover study on the influence of the chronotype in male collegiate soccer players. Chronobiol Int. 2017;34:260–268. 27906554 10.1080/07420528.2016.1256301

[7] Burn CR The polar night. Retrieved from Yukon College. Northern Research Institute 1996 [cited 2016 3 14]. Available from: https://nwtresearch.com/sites/default/files/the-polar-night.pdf

[8] Lombardi G, Vitale JA, Logoluso S, et al Circannual rhythm of plasmatic vitamin D levels and the association with markers of psychophysical stress in a cohort of Italian professional soccer players. Chronobiol Int. 2017:1–9. [Epub ahead of print]. 10.1080/07420528.2017.1297820 28306393

[9] Peiser B, Reilly T, Atkinson G, et al Seasonal changes and physiological responses: their impact on activity, health, exercise and athletic performance. Int Sportmed J. 2006;7(1):16–32.

[10] Helsedirektoratet Anbefalinger om kosthold, ernæring og fysisk aktivitet. Oslo: Helsedirektoratet. In Norwegian. (Norwegian Directory of Health (2014) Recommendations for nutrition, and physical activity.) [cited2016 3 14]Available from: http://helsedirektoratet.no/publikasjoner/anbefalinger-om-kosthold-ernering-og-fysisk-aktivitet/Publikasjoner/anbefalinger-om-kosthold-ernering-og-fysisk-aktivitet.pdf

[11] McArdle WD, Katch FI, Katch VL Exercise physiology, energy, nutrition and human performance. 3rd ed. Philadelphia: Lea & Febiger; 1991.

[12] Culnan E, Kloss JD, Grandner M A prospective study of weight gain associated with chronotype among college freshmen. Chronobiol Int. 2013;30:682–690. 23688114 10.3109/07420528.2013.782311 PMC3759532

[13] Patterson F, Malone SK, Lozano A, et al Smoking, screen-based sedentary behavior, and diet associated with habitual sleep duration and chronotype: data from the UK Biobank. Ann Behav Med. 2016;50:715–726. 27056396 10.1007/s12160-016-9797-5 PMC5079686

[14] Digdon NL, Howell AJ College students who have an eveningness preference report lower self-control and greater procrastination. Chronobiol Int. 2008;25:1029–1046. 19005903 10.1080/07420520802553671

[15] Courneya KS, Hellsten LA Personality correlates of exercise behavior, motives, barriers and preferencs: an application of the five-factor model. Personal Individ Differ. 1998;24:625–633.

[16] Vitale JA, Calogiuri G, Weydahl A Influence of chronotype on responses to a standardized, self-paced walking task in the morning vs afternoon: a pilot study. Percept Motor Skills. 2013;116:1020–1028. 24175469 10.2466/06.19.PMS.116.3.1020-1028

[17] Rossi A, Formenti D, Vitale JA, et al The effect of chronotype on psychophysiological responses during aerobic self-paced exercises. Percept Motor Skills. 2015;121:840–855. 26682609 10.2466/27.29.PMS.121c28x1

[18] Rae DE, Stephenson KJ, Roden LC Factors to consider when assessing diurnal variation in sports performance: the influence of chronotype and habitual training time-of-day. Eur J Appl Physiol. 2015;115:1339–1349. 25631930 10.1007/s00421-015-3109-9

[19] Facer-Childs E, Brandstaetter R The impact of circadian phenotype and time since awakening on diurnal performance in athletes. Curr Biol. 2015;25:1–5. 25639241 10.1016/j.cub.2014.12.036

[20] Calogiuri G, Weydahl A, Beldo S, et al Morning or evening exercise: effects on the heart rate circadian rhythm above the Arctic circle. Sport Sci Health. 2010;6:9–16.

[21] Calogiuri G, Weydahl A, Sothern RB Heart rate response to a standardized walking exercise in the Arctic circumpolar region in morning vs. evening during the polar night and midnight sun. J Sports Med Phys Fitness. 2011;51:444–451. 21904283

[22] Koukkari WL, Sothern RB Introducing biological rhythms/A Primer on the temporal organization of life, with implications for health, societies, reproduction and the natural environment. New York: Springer; 2006.

[23] Kenny W, Wilmore J, Costill D Physiology of sport and exercise. Champaign, IL: Human Kinetics; 1999.

[24] McAuley E Self-efficacy and the maintenance of exercise participation in older adults. J Behav Med. 1993;16:103–113. 8433355 10.1007/BF00844757

[25] Horne JA, Ostberg O A self-assessment questionnaire to determine morningness-eveningness in human circadian rhythms. Int J Chronobiol. 1975;4:97–110. 1027738

[26] Taillard J, Philip P, Chastang JF, et al Validation of Horne and Ostberg morningness-eveningness questionnaire in a middle-aged population of French workers. J Biol Rhythms. 2004;19:76–86. 14964706 10.1177/0748730403259849

[27] Lee JH, Kim SJ, Lee SY, et al Reliability and validity of the Korean version of morningness–eveningness questionnaire in adults aged 20–39 years. Chronobiol Int. 2014;3:479–486. 10.3109/07420528.2013.867864 PMC4031040 24467306

[28] Helsedirektoratet Fysisk form blant voksne og eldre i Norge. Resultater fra en kartlegging i 2009-2010 (is-1816). In Norwegian. (Norwegian Directory of Health (2010) Physical fitness among adults and elderly in Norway. Results from a survey in 2009–2010.) [cites 2016 3 14]. Available from: https://helsedirektoratet.no/Lists/Publikasjoner/Attachments/714/Fysisk-form-blant-voksne-og-eldre-resultater-fra-en-kartlegging-i-2009-2010-IS-1816.pdf

[29] Suni J, Ruiz JR, Castillo MJ, et al Instruments for assessing levels of physical activity and fitness (ALPHA project)., Work package 6: 53 54 55 assessing health-related fitness 12-4-2008. Amsterdam: Vrije University; 2008 [cited 2016 8 28]. Available from: https://sites.google.com/site/alphaprojectphysicalactivity/alpha-public-documents/alpha-fit/assessing-fitness-in-adults

[30] Hogben AL, Ellis J, Archer SN, et al Conscientiousness is a predictor of diurnal preference. Chronobiol Int. 2007;24:1249–1254. 18075811 10.1080/07420520701791596

[31] Giannotti F, Cortesi F, Sebastiani T, et al Circadian preference, sleep and daytime behavior in adolescence. J Sleep Res. 2002;11:191–199. 12220314 10.1046/j.1365-2869.2002.00302.x

[32] Monk TH, Buysse DJ, Potts JM, et al Morningness-eveningness and lifestyle regularity. Chronobiol Int. 2004;21:435–443. 15332448 10.1081/cbi-120038614

[33] Wittmann M, Dinich J, Merrow M, et al Social jetlag: misalignment of biological and social time. Chronobiol Int. 2006;23:497–509. 16687322 10.1080/07420520500545979

